# Serum complement C4 is an important prognostic factor for IgA nephropathy: a retrospective study

**DOI:** 10.1186/s12882-019-1420-0

**Published:** 2019-07-04

**Authors:** Tong-dan Bi, Jian-nan Zheng, Jun-xiao Zhang, Long-shu Yang, Nan Liu, Lin-lin Liu, Li Yao

**Affiliations:** Department of Nephrology, The First Affiliated Hospital of China Medical University, 155 Nan Jing North Street, He Ping District, Shen Yang, 110001 Liao Ning China

**Keywords:** Complement C4, IgA nephropathy, Biomarker, Prognosis

## Abstract

**Background:**

IgA nephropathy (IgAN) is the most common glomerulonephritis worldwide and is an important cause of end-stage renal disease (ESRD). Exploring novel biomarkers is necessary for predicting the disease activity and progression of IgAN patients. The present study sought to investigate the value of serum C4 for predicting the prognosis of IgAN patients.

**Methods:**

The primary endpoint of this retrospective study was a composite event of either a ≥ 50% reduction in estimated glomerular filtration rate (eGFR) or end-stage renal disease (ESRD) or death. The associations between serum C4 and clinicopathological parameters and prognosis of this cohort of IgAN patients were evaluated.

**Results:**

The present study included 1356 IgAN patients. Serum C4 levels correlated significantly with clinical prognostic factors. Serum C4 levels correlated positively with urinary protein excretion (*r* = 0.307, *P* < 0.001), and negatively correlated with estimated glomerular filtration rate (*r* = − 0.281, *P* < 0.001). Furthermore, serum C4 levels increased with aggravation of tubulointerstitial injury, crescents and ratios of global sclerosis (all *P* < 0.05). Prognostic analyses with the Cox proportional hazards regression model and Kaplan-Meier survival curves further identified serum C4 as an independent risk factor for the prognosis of IgAN.

**Conclusions:**

The present study identified serum C4 as a useful predictor for the prognosis of IgAN patients. The mechanism of the trend of serum C4 in IgAN needs to be illustrated in further research.

**Electronic supplementary material:**

The online version of this article (10.1186/s12882-019-1420-0) contains supplementary material, which is available to authorized users.

## Background

IgA nephropathy (IgAN) is the most common form of primary glomerulonephritis worldwide; IgAN is an important cause of end-stage renal disease (ESRD) and is not a benign condition [1–3]. The initial manifestations of IgAN are variable, ranging from asymptomatic hematuria to nephrotic syndrome or acute progressive glomerulonephritis. The hallmark of IgAN is the predominant deposition of galactose-deficient IgA1-containing immune complexes in the glomerular mesangium, and complement C3 is always present. These characteristics suggest that IgAN is an immune-mediated kidney disease, and complement activation plays an important role in the pathogenesis of IgAN [4–6].

To date, renal biopsy remains essential for making a definitive diagnosis and evaluating the prognosis of IgAN [1, 7]. However, exploring novel biomarkers is necessary for predicting the disease activity and progression of IgAN patients. Serum complement C4 has been measured widely in clinical practice for years, but its clinical significance remains uncertain for predicting the progression of IgAN. To identify the value of serum complement C4 for predicting the prognosis of IgAN patients, we performed the present study with 1356 IgAN patients and retrospectively analyzed the associations of serum complement C4 with clinicopathological parameters and prognosis of IgAN patients.

## Methods

### Study population

The present study included adult patients with primary IgAN consecutively diagnosed by renal biopsy in our department from January 2009 to December 2016. The patients with secondary IgAN were excluded, including Henoch-Schonlein purpura, ankylosing spondylitis, psoriasis, liver disease, etc. The day of renal biopsy was defined as the start day of the study. Follow-up was performed until December 2016. The patients with planned follow-up periods of greater than 1 year were further included for prognostic analyses. No corticosteroids and immunosuppressants were applied before the beginning of the study. The present study applied the recommendations outlined in the Declaration of Helsinki Principles and was approved by the Ethics Committee and the Research Board of our institution.

Serum complement C4 levels were measured by immunoturbidimetry (Beckman Coulter, Inc., USA), and the normal limit ranged from 0.16 g/L to 0.38 g/L. In addition, clinical parameters were obtained before renal biopsy, including sex, age, history of hypertension, serum levels of C3, IgA, creatinine and uric acid, 24-h urinary protein excretion (24-h UPE), and the dates of renal biopsy and follow-ups. During the periods of follow-up, data on serum creatinine, 24-h UPE, blood pressure and therapeutic regimens were also collected. The eGFR values at baseline and during follow-up periods were calculated using the Chronic Kidney Disease Epidemiology Collaboration (CKD-EPI) formula [8].

### Renal pathological evaluation

Adequate renal tissue was required for diagnostics (≥8 glomeruli in light microscopy sections and complete immunohistology and electron microscopy examination). Two pathologists evaluated histopathological manifestations separately by Oxford classifications [9, 10], including mesangial proliferation (M0/M1), endocapillary hypercellularity (E0/E1), segmental glomerulosclerosis (S0/S1), tubular atrophy/interstitial fibrosis (T0/T1/T2) and crescents (C0/C1/C2). M1 means the presence of more than 3 cells in mesangial area of > 50% of the glomeruli. E1 means the presence of an increased cells within the glomerular capillary lumina causing narrowing. S1 means the presence of any amount of the tuft involved in sclerosis. T1 means 26–50% of cortical area damaged by tubular atrophy/interstitial fibrosis, and T2 means > 50% damaged. C1 means the presence of crescent in a least 1 glomerulus, and C2 means the presence of crescents at least 25% of glomeruli. In addition, we calculated the ratios of global sclerosis.

### Treatment protocol

As previously described [11, 12], the nonimmunosuppressive therapeutic regimen was used for the IgAN patients with hematuria and/or UPE of less than 1 g/24 h and normal renal function, which included renin angiotensin system inhibitors (RASIs), anti-platelets, fish oil and statins. The immunosuppressive regimens were added for the IgAN patients with UPE of ≥1.0 g/24 h and pathological manifestations of cellular/fibrocellular crescents, moderate to severe mesangial proliferation and/or interstitial cell infiltration, including corticosteroids, cyclophosphamide, mycophenolate mofetil, leflunomide or tripterygium glycosides, applied alone or in combination.

### Definitions

The primary endpoint was a composite event of either a ≥ 50% reduction in eGFR or ESRD or death. ESRD was defined when eGFR was less than 15 ml/min/1.73 m^2^ or renal replacement therapy was initiated (i.e., hemodialysis, peritoneal dialysis or renal transplantation). Hypertension was defined as arterial blood pressures in resting state at or above 140/90 mmHg no less than twice on different days or levels less than 140/90 mmHg attained with anti-hypertensive medications.

### Statistical analyses

Skewed distributed continuous variables were expressed as median and interquartile range and compared with the nonparametric test. Normally distributed continuous variables were expressed as the means ± SD and compared with the T test, and categorical variables were expressed as absolute frequencies and percentages and compared with the chi-square test. Pearson’s correlation was applied to analyze the association of serum C4 with age, 24-h UPE, eGFR, IgA and complement C3, and ratios of global sclerosis. To compare serum C4 levels between different grades of pathological parameters, nonparametric tests were performed. To identify the independent prognostic value of serum C4, a Cox proportional hazards regression model was applied for univariable and multivariable analyses using the “Enter” method. Furthermore, we stratified the levels of serum C4 into three categories: low (< 0.16 g/L), normal (0.16 to 0.38 g/L) and high (> 0.38 g/L). Kaplan-Meier survival analysis was performed to estimate the discriminative ability of serum C4 for predicting the prognosis of IgAN patients.

All the *P*-values were two-tailed. *P* < 0.05 was considered statistically significant. All the analytic procedures were performed with SPSS version 16.0 (SPSS, Inc., Chicago, IL, USA).

## Results

### Characteristics of the included IgAN patients

We included 1356 adult IgAN patients. The baseline clinicopathological characteristics were described in Table 1. The original data were supplied in Additional file 2. The mean age was 37 ± 13 years old. In total, 49.9% of patients were males. The average levels of eGFR and 24-h UPE were 83.85 ± 34.19 ml/min/1.73 m^2^ and 2.24 ± 2.50 g/d, respectively. In total, 41% of patients were hypertensive. Furthermore, 1052 patients had follow-up periods of more than 1 year and complete information and were included for prognostic analyses. No patients were treated with immunosuppressive agents before the start of the study, but 72.1 and 73.3% of the patients in the primary and follow-up cohorts were treated with immunosuppressive agents during the periods of follow-up, respectively. No significantly different variables were found between the primary and the follow-up cohorts (all *P* > 0.05).

**Table 1:** Univariate and multivariable Cox proportional hazards regression analysis of the data from the development cohort (the composite endpoint)

Predictors	Univariable analysis			Multivariable analysis		
	HR	95%CI	*P* value	HR	95%CI	*P* value
Sex			< 0.001			0.664
Male	1.000			1.000		
female	0.514	0.365, 0.725	< 0.001	1.096	0.724, 1.658	0.664
Age at renal biopsy	1.018	1.006, 1.031	0.005	0.978	0.962, 0.994	0.007
Hypertension			< 0.001			0.572
No	1.000			1.000		
Yes	4.009	2.804, 5.731	0.001	1.132	0.735, 1.743	0.572
UPE (g/d)	1.199	1.162, 1.237	< 0.001	1.061	1.005, 1.120	0.033
eGFR (ml/min/1.73m^2^)	0.947	0.940, 0.953	< 0.001	0.960	0.950, 0.970	< 0.001
Serum uric acid	1.006	1.005, 1.007	< 0.001	1.001	1.000, 1.003	0.126
Serum IgA	0.830	0.713, 0.966	0.016	0.895	0.760, 1.054	0.182
Serum C3	2.197	1.113, 4.338	0.023	2.393	0.942, 6.080	0.067
Serum C4	902.010	285.539, 2576.250	< 0.001	6.978	1.013, 48.070	0.048
RASI			< 0.001			0.341
Not received	1.000			1.000		
Received	0.274	0.196, 0.383	< 0.001	1.231	0.802, 1.890	0.341
Immunosuppressants			0.258	–	–	–
Not received	1.000					
Received	1.255	0.847, 1.859	0.258			
Oxford classification						
Mesangial hypercellularity			< 0.001			0.031
M0	1.000			1.000		
M1	3.056	1.898, 4.918	< 0.001	1.847	1.057, 3.227	0.031
Endocapillary hypercellularity			0.451	–	–	–
E0	1.000					
E1	0.865	0.593, 1.262	0.451			
Segmental sclerosis			< 0.001			0.124
S0	1.000					
S1	4.009	2.804, 5.731	< 0.001	0.723	0.479, 1.093	0.124
Tubular atrophy/interstitial fibrosis			< 0.001			0.048
T0	1.000			1.000		
T1	6.578	3.551, 12.185	< 0.001	1.641	0.829, 3.250	0.155
T2	35.612	20.203, 62.772	< 0.001	2.480	1.175, 5.236	0.017
Crescents			< 0.001			0.031
C0				1.000		
C1	1.430	0.996, 2.053	0.053	1.384	0.924, 2.073	0.114
C2	2.909	1.824, 4.641	< 0.001	2.133	1.206, 3.770	0.009
Glomerulosclerosis	87.864	49.586, 155.690	< 0.001	3.734	1.528, 9.127	0.004

*UPE* urinary protein excretion, *eGFR* estimated glomerular filtration rate, *RASI* renin-angiotensin system inhibitors

### Correlations between serum C4 and clinical parameters

**Fig. 1:**
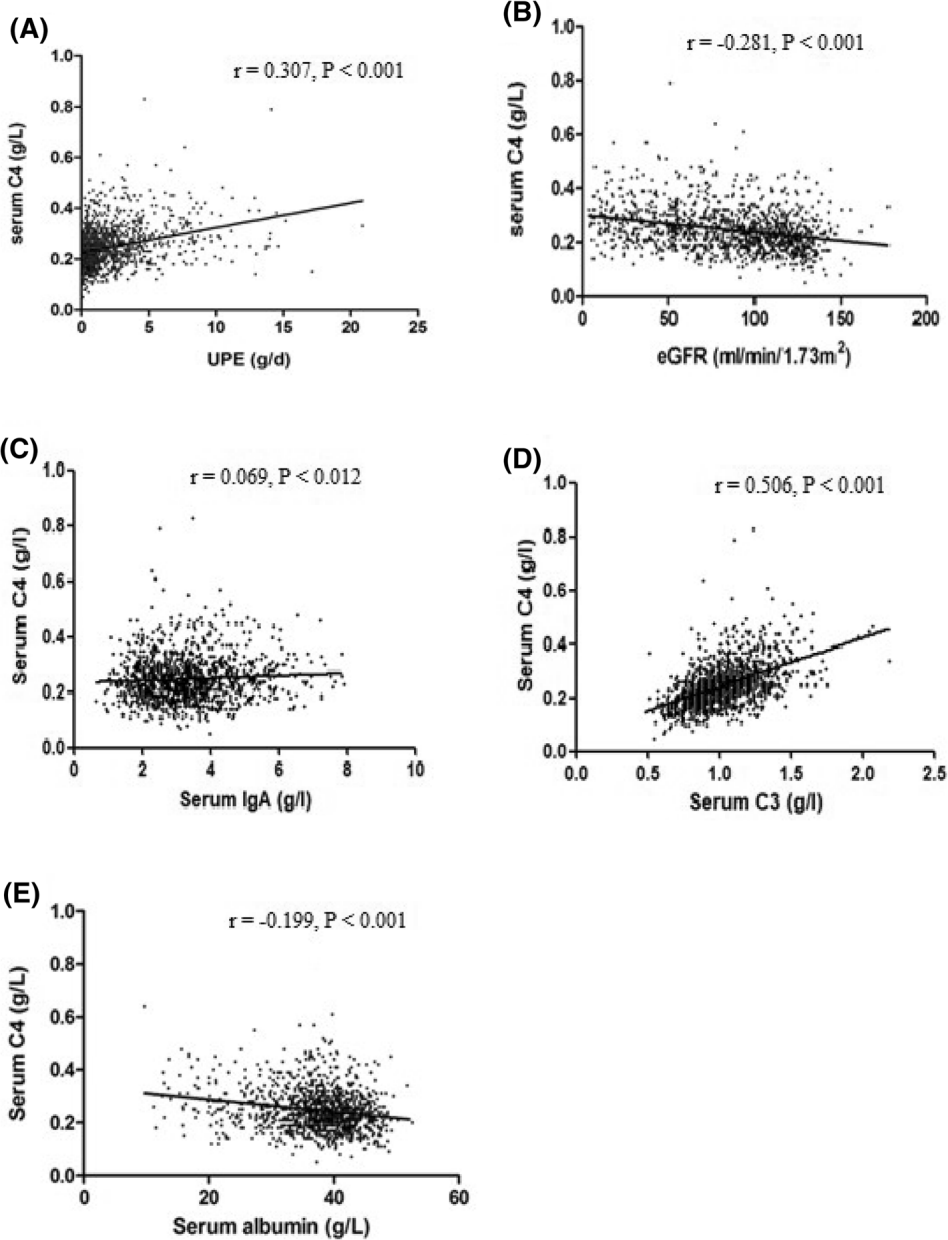
Correlations between serum C4 and clinical parameters, including 24-h urinary protein excretion (UPE) (**a**), estimated glomerular filtration rate (**b**), serum IgA (**c**), serum C3 (**d**) and serum albumin (**e**)

First, serum C4 levels were significantly higher in male patients (0.25, IQR 0.20–0.30) than female patients (0.23, IQR 0.18–0.27) (*P* < 0.001) and correlated positively with age (*r* = 0.163, *P* < 0.001). Serum C4 levels correlated positively with the levels of 24-h UPE (*r* = 0.307, *P* < 0.001, Fig. 1a) but negatively correlated with eGFR (*r* = − 0.281, *P* < 0.001, Fig. 1b). Furthermore, serum C4 levels were positively correlated with IgA (*r* = 0.069, *P* < 0.012, Fig. 1c) and C3 (*r* = 0.506, *P* < 0.001, Fig. 1d) but negatively correlated with albumin serum levels (*r* = − 0.199, *P* < 0.001, Fig. 1e).

### Correlations between serum C4 and histopathological parameters

**Fig. 2:**
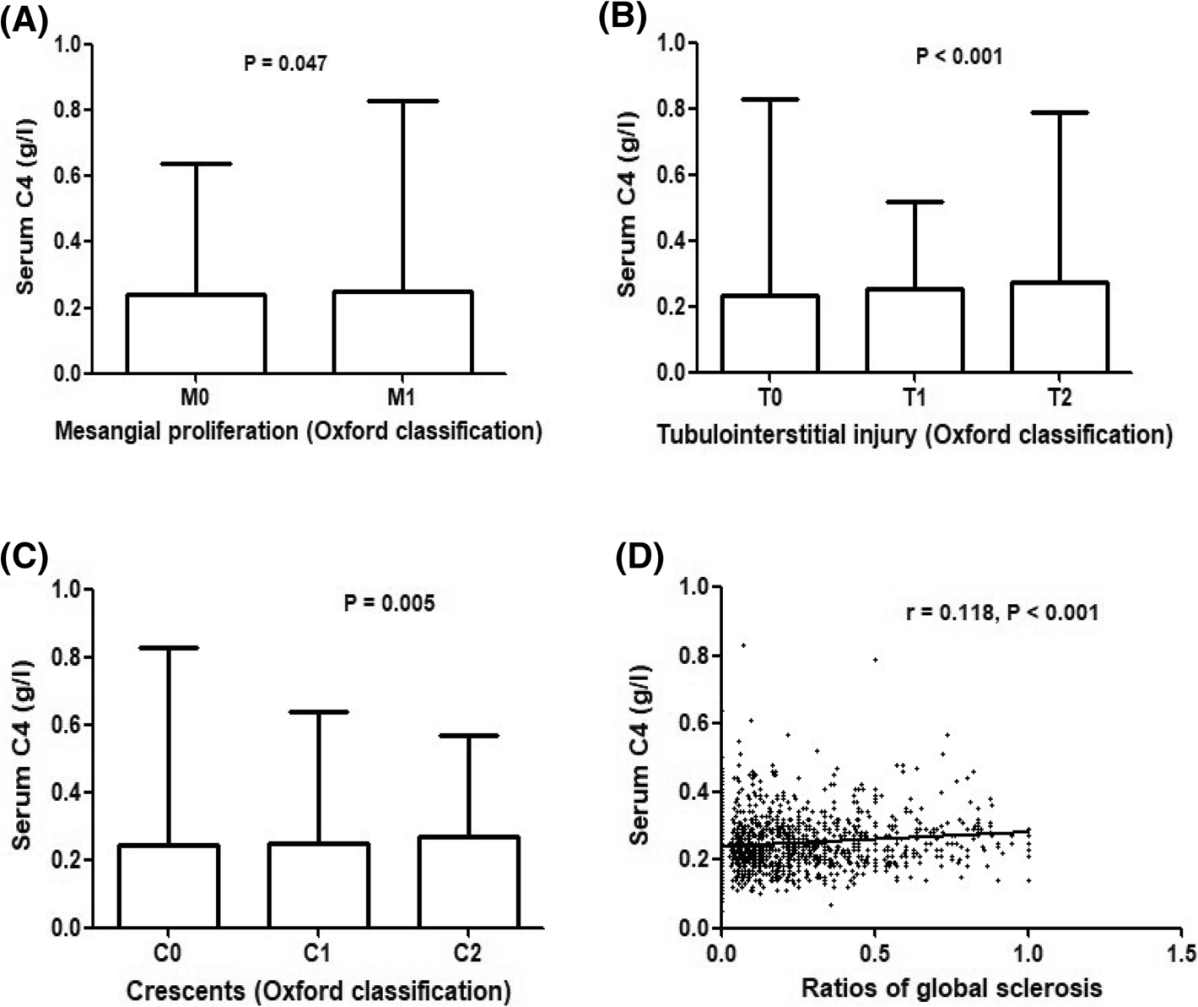
Correlations between serum complement C4 and histopathological parameters, including mesangial proliferation (**a**), tubulointerstitial injury (**b**), crescents (**c**) and ratios of global sclerosis (**d**)

For mesangial proliferation, patients with M1 had significantly higher levels of serum C4 (0.24, IQR 0.20–0.29) than those without M0 (0.23, IQR 0.18–0.28) (*P* = 0.047) (Fig. 2a). Serum C4 levels were not associated with segmental glomerulosclerosis or endocapillary hypercellularity. Serum C4 increased in parallel to the severity of tubule-interstitial lesions (T0, 0.22, IQR 0.18–0.27; T1, 0.25, IQR 0.20–0.30; T2, 0.26, IQR 0.21–0.31; *P*_0,1_ < 0.001, *P*_1,2_ = 0.057, *P*_0,2_ < 0.001) (Fig. 2b).

For crescent formation, serum C4 levels were significantly higher in the patients with C2 than those in the patients with C1 and C0 (C0, 0.24 IQR 0.19–0.29; C1, 0.23, IQR 0.19–0.28; C2, 0.27, IQR 0.21–0.31, *P*_0,1_ = 0.466, *P*_1,2_ = 0.001, *P*_0,2_ = 0.004) (Fig. 2c). In addition, serum C4 levels were positively correlated with the ratios of global sclerosis (*r* = 0.118, *P* < 0.001) (Fig. 2d).

### Correlation between serum C4 levels and prognosis

To investigate the association between serum C4 levels and the composite endpoint, we analyzed the data of the 1157 patients with the planned follow-up periods of more than 1 year. The mean follow-up period was 48 ± 23 months. In total, 143 patients (13.59%) experienced the composite endpoint, including 103 patients (9.79%) progressing to ESRD, 31 patients (2.95%) with a ≥ 50% reduction in eGFR, and 9 deaths (0.86%).

**Fig. 3:**
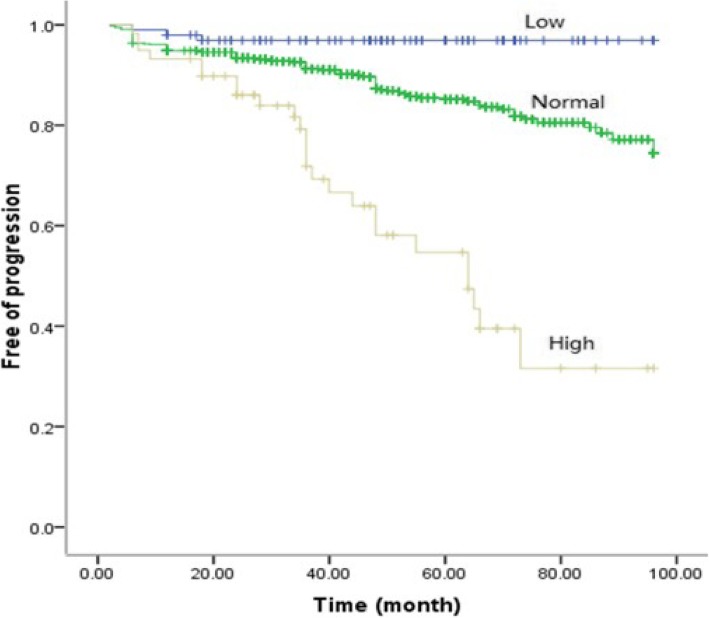
The predictive value of serum C4 in IgAN progression (Kaplan-Meier survival curves)

Univariable analysis of the Cox proportional hazards regression model identified the following variables for multivariable analysis: sex, age, history of hypertension, UPE, estimated glomerular filtration rate (eGFR), serum uric acid, IgA, C3, C4, treatment with RASIs, mesangial hypercellularity, segmental sclerosis, tubular atrophy/interstitial fibrosis, crescents and ratios of global sclerosis (Table 1). Multivariable analysis further verified serum C4 as an independent risk factor for the composite endpoint of IgAN [hazard ratio (HR) 6.978, 95% confidence interval (CI), 1.013–48.070, *P* = 0.048)] (Table 1) and for the endpoint of 50% decrease of eGFR (HR 547.272, 95% CI 12.030–24,895.764, *P* = 0.001) (Additional file 1: Table S1), but not for ESRD and death (Additional file 1: Table S2 and S3). The ratios of low, normal and high serum C4 groups were 9.32, 85.08 and 5.61%, respectively. Kaplan-Meier survival curves showed good discrimination of serum C4 for predicting the prognosis of IgAN patients (Fig. 3).

## Discussion

The present study with 1356 IgAN patients showed that serum complement C4 was an important prognostic factor for IgAN. First, serum C4 levels correlated with clinical prognostic factors. Specifically, serum C4 levels positively correlated with UPE but negatively correlated with eGFR and serum albumin. Second, serum C4 levels positively correlated with aggravation of mesangial proliferation, tubulointerstitial injury, crescents and ratios of glomerulosclerosis. The prognostic analyses identified serum C4 as an independent risk factor of IgAN progression.

In recent years, novel biomarkers had attracted wide attention for diagnosis and predicting prognosis in IgAN [13, 14], particularly biomarkers based on the pathogenesis [15–17]. The present study demonstrated that high serum C4 levels were correlated with severe clinical and pathological manifestations, and serum C4 was further identified as an independent risk factor for the progression of IgAN. Although the ratio of high serum C4 group is only 5.61%, the correlation between serum C4 levels and the progression of IgAN remained even when the serum C4 levels were nomral. So we believed that serum C4 is a good predictor. Notably, serum complement C4 is easily detected and is expected to be used widely in clinical practice.

A previous study demonstrated that the IgAN patients with low C4 levels might be associated with a poor prognosis; however, they exhibited better renal presentations at the time of renal biopsy [18]. Equivocal results may be associated with the limited sample size (*n* = 512) and the high lost to follow-up rate (53.1%). The present study included 1356 IgAN patients, and the lost to follow-up rate was only 9.08%, which verified the reliability of our conclusions.

However, the mechanism explaining how increased serum C4 is related to the poor prognosis of IgAN remains unclear. Complement components are well-known acute phase proteins, including C3 and C4 [19, 20]. In the present study, the parameters associated with serum C4 were all related to the conditions with more inflammation, such as higher proteinuria, lower eGFR, increased tubular atrophy and lower albumin predictive value of serum C4 was independent of these factors. These results may indicate the role of serum C4 in IgAN as an acute phase protein.

On the other hand, complement C4 is also an important factor involved in the activation of complement pathways. We previously reported the association between C4d deposition and clinicopathological manifestations and prognosis in a cohort of 131 IgAN patients [12]. In this cohort, 23.08% exhibited C4 deposition, and patients with C4 deposition had higher levels of serum C4 than those without C4 deposition (0.27 ± 0.08 vs. 0.23 ± 0.08, *P* = 0.011). We also previously reported the association between MBL deposition and clinicopathogical manifestations in a cohort of 165 IgAN patients [21]. In this cohort, 2 patients had no serum C4 results, so we analyzed the results of the remaining 163 patients. Patients with MBL deposition had higher serum C4 levels compared with those without C4 deposition, but the difference was not statistically significant (0.25 ± 0.08 vs. 0.23 ± 0.07, *P* = 0.078). These results indicated that increased serum C4 might be associated with the activation of lectin complement pathway in IgAN.

It has been acknowledged that complement C4 was involved in the activation of classical and lectin pathways. The previous study showed that classical pathway activation was not involved in IgAN [22, 23]. In recent years, increasing evidence suggests that the lectin pathway is involved in IgAN [11, 12, 24]. As previously noted, we have confirmed that glomerular deposition of mannose binding lectin and C4d was correlated with severe clinicopathological manifestations and poor prognosis of IgAN [12, 21], indicating that lectin pathway activation in the kidney may lead to more severe renal lesions in IgAN. However, given the formation of immune complexes in circulation [25, 26], we hypothesized that activation of the complement pathway could also occur in circulation of IgAN patients, which may also play a harmful role in IgAN progression.

However, the present study had some limitations. First, the large time span and the discrepancy of therapeutic regimens may affect the results of our study. Second, as a single-center study, our study could not exclude the limits of races and regions, so its external validity may be limited. Finally, we did not investigated the mechanisms of the increase of serum C4 in the progression of IgAN, which should be explored in future research.

## Conclusions

In summary, we concluded that serum C4 levels were significantly correlated with the well-acknowledged clinicopathological prognostic factors of IgAN, and serum C4 may be an independent risk factor for IgAN progression. The present study may indicate the harmful role of complement activation in IgAN circulation. However, the mechanism of the trend of serum C4 in IgAN must be illustrated in further research.

## Additional files

Additional file 1: **Table S1.** Univariate and multivariable Cox proportional hazards regression analysis of the data from development cohort (≥50% reduction of eGFR). **Table S2.** Univariate and multivariable Cox proportional hazards regression analysis of the data from development cohort (ESRD). **Table S3.** Univariate and multivariable Cox proportional hazards regression analysis of the data from development cohort (death). (DOCX 37 kb)

Additional file 2. Data. (XLSX 509 kb)

## Data Availability

The datasets used and/or analyzed during the present study are available from the corresponding author on reasonable request.

## Abbreviations

CKD-EPI: Chronic Kidney Disease Epidemiology Collaboration
eGFR: estimated glomerular filtration rates
ESRD: End-stage renal disease
IgAN: IgA nephropathy
RASI: Renin angiotensin system inhibitors;
SD: Standard deviation
UPE: Urinary protein excretion
