# Supplementary material for: Serum complement C4 is an important prognostic factor for IgA nephropathy: a retrospective study
Source: BMC Nephrol. 2019 Jul 4;20:244. doi: 10.1186/s12882-019-1420-0 (PMC6610919; doi:10.1186/s12882-019-1420-0)
Supplement: Supplementary file 1 — Table S1. Univariate and multivariable Cox proportional hazards regression analysis of the data from development cohort (≥50% reduction of eGFR). Table S2. Univariate and multivariable Cox proportional hazards regression analysis of the data from development cohort (ESRD). Table S3. Univariate and multivariable Cox proportional hazards regression analysis of the data from development cohort (death). (DOCX 37 kb) [file 12882_2019_1420_MOESM1_ESM.docx]

Table S1. Univariate and multivariable Cox proportional hazards regression analysis of the data from development cohort (≥50% reduction of eGFR)

| Predictors | Univariable analysis | | |  | Multivariable analysis | | |
| --- | --- | --- | --- | --- | --- | --- | --- |
|  | HR | 95%CI | P value |  | HR | 95%CI | P value |
| Sex  Male  female  Age at renal biopsy  Hypertension  No  Yes  UPE (g/d)  eGFR (ml/min/1.73m^2^)  Serum uric acid  Serum IgA  Serum C3  Serum C4  RASI  Not received  Received  Immunosuppressants  Not received  Received | 1.000  0.528  1.022  1.000  1.717  1.138  0.981  1.005  0.840  13.998  2594.450  1.000  1.200  1.000  1.483 | 0.253, 1.101  0.994, 1.050  0.849, 3.475  1.049, 1.234  0.971, 0.992  1.002, 1.007  0.603, 1.171  4.259, 46.011  340.629, 19760.981  0.537, 2.684  0.608, 3.617 | 0.089  0.089  0.120  0.133  0.133  0.002  0.001  0.001  0.305  <0.001  <0.001  0.657  0.657  0.386  0.386 |  | 1.000  0.986  —  1.000  1.019  0.944  1.009  1.003  0.871  10.292  547.272  1.000  1.530  — | 0.605, 1.605  —  0.598, 1.737  0.808, 1.102  0.993, 1.026  1.000, 1.007  0.718, 1.057  1.982, 53.454  12.030, 24895.764  0.897, 2.609  — | 0.954  0.954  —  0.945  0.945  0.463  0.260  0.069  0.163  0.006  0.001  0.118  0.118  — |

(To be continued)

Table S1 (continued). Univariate and multivariable Cox proportional hazards regression analysis of the data from development cohort (≥50% reduction of eGFR)

| Predictors | Univariable analysis | | |  | Multivariable analysis | | |
| --- | --- | --- | --- | --- | --- | --- | --- |
|  | HR | 95%CI | P value |  | HR | 95%CI | P value |
| Oxford classification  Mesangial hypercellularity  M0  M1  Endocapillary hypercellularity  E0  E1  Segmental sclerosis  S0  S1  Tubular atrophy/interstitial fibrosis  T0  T1  T2  Crescents  C0  C1  C2  Glomerulosclerosis | 1.000  15.875  1.000  1.061  1.000  2.671  1.000  4.782  8.059  1.000  1.252  3.805  8.122 | 2.161, 116.611  0.488, 2.305  1.250, 5.709  1.929, 11.854  3.056, 21.250  0.562, 2.787  1.497, 9.671  1.829, 36.068 | 0.007  0.007  0.882  0.882  0.011  0.011  <0.001  0.001  <0.001  0.015  0.582  0.005  0.006 |  | 1.000  10.723  —  1.000  1.195  1.000  2.455  3.605  1.000  0.951  2.257  1.065 | 1.407, 81.749  —  0.518, 2.760  0.847， 7.122  0.827， 15.706  0.413, 2.189  0.768, 6.632  0.099, 11.448 | 0.022  0.022  —  0.676  0.676  0.193  0.098  0.088  0.228  0.906  0.139  0.959 |

UPE, urinary protein excretion; eGFR, estimated glomerular filtration rate; RASI, renin-angiotensin system inhibitors.

Table S2. Univariate and multivariable Cox proportional hazards regression analysis of the data from development cohort (ESRD)

| Predictors | Univariable analysis | | |  | Multivariable analysis | | |
| --- | --- | --- | --- | --- | --- | --- | --- |
|  | HR | 95%CI | P value |  | HR | 95%CI | P value |
| Sex  Male  female  Age at renal biopsy  Hypertension  No  Yes  UPE (g/d)  eGFR (ml/min/1.73m^2^)  Serum uric acid  Serum IgA  Serum C3  Serum C4  RASI  Not received  Received  Immunosuppressants  Not received  Received | 1.000  0.477  1.009  1.000  5.797  1.211  0.926  1.007  0.783  1.102  498.488  1.000  0.188  1.000  1.263 | 0.316, 0.720  0.993, 1.024  3.644, 9.222  1.168, 1.255  0.916, 0.937  1.006, 1.008  0.652, 0.941  0.470, 2.585  112.076, 2217.164  0.124, 0.286  0.794, 2.009 | <0.001  0.705  0.277  <0.001  <0.001  <0.001  <0.001  <0.001  0.009  0.823  <0.001  <0.001  <0.001  0.325  0.325 |  | 1.000  0.986  —  1.000  1.019  1.097  0.941  1.002  0.871  —  3.963  1.000  1.530  — | 0.605, 1.605  —  0.598, 1.737  1.029, 1.169  0.926, 0.956  1.000, 1.004  0.718, 1.057  —  0.485, 32.385  0.897, 2.609  — | 0.954  0.954  —  0.945  0.945  0.005  <0.001  0.028  0.163  —  0.199  0.118  0.118  — |

(To be continued)

Table S2 (continued). Univariate and multivariable Cox proportional hazards regression analysis of the data from development cohort (ESRD)

| Predictors | Univariable analysis | | |  | Multivariable analysis | | |
| --- | --- | --- | --- | --- | --- | --- | --- |
|  | HR | 95%CI | P value |  | HR | 95%CI | P value |
| Oxford classification  Mesangial hypercellularity  M0  M1  Endocapillary hypercellularity  E0  E1  Segmental sclerosis  S0  S1  Tubular atrophy/interstitial fibrosis  T0  T1  T2  Crescents  C0  C1  C2  Glomerulosclerosis | 1.000  2.256  1.000  0.789  1.000  2.000  1.000  10.270  85.925  1.000  1.408  2.378  214.114 | 1.349, 3.773  0.500, 1.247  1.328, 3.012  3.845, 27.430  34.514, 213.915  0.924, 2.146  1.334, 4.239  107.678, 425.759 | 0.002  0.002  0.311  0.311  0.001  0.001  <0.001  <0.001  <0.001  0.012  0.111  0.003  <0.001 |  | 1.000  1.202  —  1.000  0.694  1.000  2.829  4.377  1.000  1.540  2.124  6.318 | 0.650, 2.225  —  0.420, 1.149  0.914， 8.754  1.373， 13.951  0.941, 2.520  1.023, 4.410  2.273, 17.560 | 0.557  0.557  —  0.156  0.156  0.040  0.071  0.013  0.085  0.086  0.043  <0.001 |

ESRD, end-stage renal disease; UPE, urinary protein excretion; eGFR, estimated glomerular filtration rate; RASI, renin-angiotensin system inhibitors.

Table S3. Univariate and multivariable Cox proportional hazards regression analysis of the data from development cohort (death)

| Predictors | Univariable analysis | | |  | Multivariable analysis | | |
| --- | --- | --- | --- | --- | --- | --- | --- |
|  | HR | 95%CI | P value |  | HR | 95%CI | P value |
| Sex  Male  female  Age at renal biopsy  Hypertension  No  Yes  UPE (g/d)  eGFR (ml/min/1.73m^2^)  Serum uric acid  Serum IgA  Serum C3  Serum C4  RASI  Not received  Received  Immunosuppressants  Not received  Received | 1.000  0.776  1.129  1.000  3.501  1.247  0.916  1.001  1.417  0.787  2996.156  1.000  0.054  1.000  0.702 | 0.208, 2.889  1.071, 1.190  0.874, 14.026  1.127, 1.380  0.877, 0.956  0.995, 1.007  0.876, 2.294  0.040, 15.608  60.902, 147399.353  0.007, 0.433  0.175, 2.808 | 0.705  0.705  <0.001  0.077  0.077  <0.001  <0.001  0.781  0.156  0.875  <0.001  0.006  0.006  0.616  0.616 |  | —  1.073  —  1.148  0.933  —  —  —  46.163  1.000  0.456  — | —  1.011, 1.139  —  0.965, 1.366  0.883, 0.985  —  —  —  0.086, 24873.29  0.031, 6.617  — | —  0.020  —  0.120  0.013  —  —  —  0.232  0.565  0.565  — |

(To be continued)

Table S3 (continued). Univariate and multivariable Cox proportional hazards regression analysis of the data from development cohort (death)

| Predictors | Univariable analysis | | |  | Multivariable analysis | | |
| --- | --- | --- | --- | --- | --- | --- | --- |
|  | HR | 95%CI | P value |  | HR | 95%CI | P value |
| Oxford classification  Mesangial hypercellularity  M0  M1  Endocapillary hypercellularity  E0  E1  Segmental sclerosis  S0  S1  Tubular atrophy/interstitial fibrosis  T0  T1  T2  Crescents  C0  C1  C2  Glomerulosclerosis | 1.000  4.177  1.000  1.270  1.000  4.579  1.000  7.385  27.404  4.943  24.068  3.079 | 0.517, 33.770  0.317, 5.090  0.938, 22.345  0.767, 71.086  3.155, 238.021  0.552, 44.227  2.686, 215.660  0.170, 55.673 | 0.180  0.180  0.736  0.736  0.060  0.060  0.006  0.084  0.003  0.007  0.153  0.004  0.446 |  | —  —  —  1.000  2.190  1.034  1.000  5.668  6.970  — | —  —  —  0.145， 33.066  0.099， 10.770  0.521, 61.634  0.727, 66.807  — | —  —  —  0.701  0.571  0.978  0.228  0.154  0.092  — |

UPE, urinary protein excretion; eGFR, estimated glomerular filtration rate; RASI, renin-angiotensin system inhibitors.
